# Heteroplasmy Is Rare in Plant Mitochondria Compared with Plastids despite Similar Mutation Rates

**DOI:** 10.1093/molbev/msae135

**Published:** 2024-06-27

**Authors:** Marina Khachaturyan, Mario Santer, Thorsten B H Reusch, Tal Dagan

**Affiliations:** Marine Evolutionary Ecology, GEOMAR Helmholtz Centre for Ocean Research Kiel, Kiel, Germany; Institute of General Microbiology, University of Kiel, Kiel, Germany; Institute of General Microbiology, University of Kiel, Kiel, Germany; Marine Evolutionary Ecology, GEOMAR Helmholtz Centre for Ocean Research Kiel, Kiel, Germany; Institute of General Microbiology, University of Kiel, Kiel, Germany

**Keywords:** plant organelle evolution, plastids, mitochondria, eelgrass, substitution rate, genetic diversity, allele dynamics, heteroplasmy, simulated evolution, *Zostera marina*

## Abstract

Plant cells harbor two membrane-bound organelles containing their own genetic material—plastids and mitochondria. Although the two organelles coexist and coevolve within the same plant cells, they differ in genome copy number, intracellular organization, and mode of segregation. How these attributes affect the time to fixation or, conversely, loss of neutral alleles is currently unresolved. Here, we show that mitochondria and plastids share the same mutation rate, yet plastid alleles remain in a heteroplasmic state significantly longer compared with mitochondrial alleles. By analyzing genetic variants across populations of the marine flowering plant *Zostera marina* and simulating organelle allele dynamics, we examine the determinants of allele segregation and allele fixation. Our results suggest that the bottlenecks on the cell population, e.g. during branching or seeding, and stratification of the meristematic tissue are important determinants of mitochondrial allele dynamics. Furthermore, we suggest that the prolonged plastid allele dynamics are due to a yet unknown active plastid partition mechanism. The dissimilarity between plastid and mitochondrial novel allele fixation at different levels of organization may manifest in differences in adaptation processes. Our study uncovers fundamental principles of organelle population genetics that are essential for further investigations of long-term evolution and molecular dating of divergence events.

## Introduction

Genetics of the eukaryotic organelles—plastid and mitochondria—share multiple characteristics with the genetics of other extrachromosomal genetic elements (ECEs). For example, the organelle genome copy number may significantly outnumber that of the nuclear chromosomes, similar to prokaryotic plasmids (reviewed in Rodríguez-Beltrán et al. 2021). The high copy number entails the possibility of intracellular genetic diversity, termed “heteroplasmy” (reviewed in Ramsey and Mandel 2019). The intermediate intracellular allele frequency (AF) changes due to random allele segregation during the organelle and cell division, termed “segregational drift” (Birky et al. 1989). Segregational drift of plastid alleles occurs independently during plastid and cell division. The effect of mitochondrial division on allele segregation is considered negligible due to regular mitochondrial fusion and fission (Lonsdale et al. 1988; Logan 2010; Rose 2019). Plastids and mitochondria are typically considered to be uniparentally inherited, in contrast to the biparentally inherited nuclear genome, although organelle paternal (and maternal) leakage was previously reported (reviewed in Ramsey and Mandel 2019 and Camus et al. 2022). Assuming strict uniparental inheritance, organelles evolve analogous to asexual microbes, with new variants arising solely via de novo mutations. Consequently, organelle genomes are expected to accumulate slightly deleterious mutations over time, eventually leading to mutational meltdown (Gabriel et al. 1993). The main factor that mitigates mutational meltdown in plant organelles is thought to be their lower mutation rate compared with the nuclear genome (Palmer and Herbon 1988; Lynch et al. 2006). Additionally, interchromosomal recombination (gene conversion) was suggested to prevent the organelle mutational meltdown (Khakhlova and Bock 2006; Maréchal and Brisson 2010; Edwards et al. 2021).

The maintenance of organelles upon cell division requires their even partition into daughter cells. Prior to cell cytokinesis, mitochondrial fission into small entities that are unbiasedly scattered in the cytoplasm (Sheahan et al. 2004). The number of these small mitochondria may exceed the mitochondrial DNA (mtDNA) copy number (Arimura et al. 2004; Preuten et al. 2010). An even cytoplasm volume division between the two daughter cells thereby ensures that the mtDNA distribution is balanced (Sheahan et al. 2004, 2007). In contrast, plastids are localized in the perinuclear area prior to the cell division, where the number of plastids is doubled by plastid division. During cytokinesis, plastids cluster around the daughter nuclei, which may ensure their segregation. The proper plastid positioning throughout the cell cycle is ensured by actin filaments (Sheahan et al. 2004). However, how plastids are evenly partitioned into the daughter cells remains unclear.

In multicellular organisms, the accumulation of mutations in nuclear and organelle genomes is slowed down within the germline (Milholland et al. 2017). Recent studies suggest that few slowly dividing pluripotent stem cells in the shoot apical meristem (SAM) central zone—termed “shoot apical initials” and referred to as “stem cells”—later form a transient sexually active plant germline (Kwiatkowska 2008; Lanfear 2018; Burian 2021). The stem cell population undergoes regular bottlenecks during stem branching and seed formation. Stem cells divide preferentially within the corresponding meristematic layer, which comprises one to two tunica layers and an internal corpus layer. Most cell divisions of stem cells are asymmetrical, i.e. only one daughter cell remains a stem cell while the other one initiates the differentiation pathway. Symmetrical cell division yields two daughter stem cells; cell differentiation eventually preserves the total number of stem cells on the apex (Bowman and Eshed 2000; Morrison and Kimble 2006; Burian 2021). The effect of the plant meristem structure on the organelle DNA (oDNA) allele dynamics remains understudied.

As our model species, we chose the seagrass species *Zostera marina* (eelgrass), a widespread marine-flowering plant that radiated across the Atlantic and Pacific Oceans several hundred thousand years ago (Yu et al. 2023). Eelgrass reproduces both sexually and vegetatively by branching of rhizomes, which leads to large eelgrass meadow formation by ramets of the same clone that can be several hundred years old (Reusch et al. 1999). The rhizomes involved in ramet initiation are formed from the SAM (Marbà and Duarte 1998). Eelgrass ramets, unlike tree branches, separate from their parental plants and therefore are susceptible to selection within a single clone (Yu et al. 2020). The availability of the nuclear, plastid, and mitochondrial genome assembly and meristem imaging, together with the standardized worldwide population DNA sequencing data set, makes *Z. marina* an interesting species for a short-term organelle evolution study (Olsen et al. 2016; Ma et al. 2021; Khachaturyan et al. 2023; Yu et al. 2023, 2024).

**Fig. 1.**
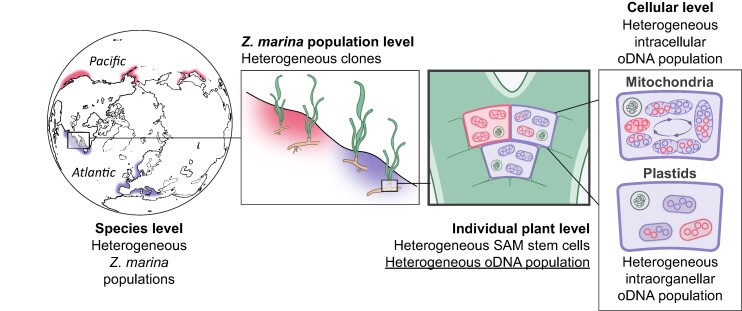
Heterogeneous populations at different levels of biological organization of *Z. marina*. The scheme illustrates populations at different levels of biological organization including (left to right) plants, cells within a plant, and organelles within a cell. Genetic diversity and allele homo- and heterogeneity manifest in these populations at different levels of organization. Due to the regular mitochondrial fusion and fission, the mtDNA population cannot be defined at the deepest intraorganellar level in contrast to the ptDNA population. The bold frame delineates the main level of biological organization on which this study is focused.

Here, we used the available organellar DNA data of *Z. marina* from meristematic regions sampled from individual plants to infer the neutral substitution rates and frequency of intraindividual heteroplasmy in mitochondria and plastids. We then used computer simulations to estimate the effect of individual properties of the plant organelle biology on the segregation dynamics of newly emerging neutral alleles. For the purpose of our study, several definitions are required as the term “population” refers to different levels of biological organizations. Primarily, we study allele dynamics in populations of oDNA molecules—mtDNA or plastid DNA (ptDNA)—in a set of SAM stem cells (Fig. 1). Therefore, fixation of a certain allele in the focal oDNA population (i.e. substitution) corresponds to allele fixation at the level of an individual plant, while the population of plants may remain heterogeneous. Accordingly, substitution rates are estimated from all fixation events in one individual plant. We refer to the organelle population in SAM stem cells of one plant as an oDNA, mtDNA, or ptDNA population. Additionally, when describing processes that manifest at the cellular level, we denote the set of SAM stem cells as the “cell population.” For conventional populations of individual plants and clones, we use the term “eelgrass population” or, alternatively, specify their geographical location, e.g. “Alaskan population.” With these definitions, we delineate the effect of processes at different levels of biological organizations on allele dynamics, with a primary focus on oDNA populations (Fig. 1).

## Results

### The Nucleotide Substitution Rate in the *Z. marina* Organelle Genomes Is Homogeneous

To compare the rate of evolution between the two plant organelles, we detected single nucleotide polymorphisms (SNPs) in mitochondrial and plastid genomes across 110 samples from 9 eelgrass populations residing in the Atlantic Ocean and 2 populations residing near the Alaskan coast. These populations were specifically selected for the analysis since the inference of their divergence time is considered reliable due to their geographical separation (Yu et al. 2023). We focused only on neutral positions (here defined as intergenic positions), in which no SNP detection bias is expected, e.g. by excluding nuclear mitochondrial (NUMT), nuclear plastid (NUPT), and mitochondrial plastid DNA (mtptDNA; Kleine et al. 2009; Scarcelli et al. 2016). The detection of SNPs at neutral positions within the mitochondrial genomes revealed 33 to 80 SNPs that separate the Atlantic and the Alaskan mitogenomes, further referred to as “genetic distance” (supplementary fig. S1, Supplementary Material online). The number of SNPs was, on average, 57, which corresponds to an average of 28.5 mutations accumulated since the divergence of the Atlantic and the Alaskan populations, which was estimated to occur 243 kya (Yu et al. 2023). Thus, the total of 28.5 accumulated mutations corresponds to a substitution rate of $9.6\times 10^{-10}$ substitutions per base pair per year (Table 1). The SNPs at neutral plastid positions revealed a genetic distance of 20 to 26 SNPs between the Atlantic and the Alaskan plastid genomes (supplementary fig. S2, Supplementary Material online). The genetic distance was 23 SNPs on average, which corresponds to an average of 11.5 accumulated mutations since the divergence time; 11.5 plastid mutations correspond, thereby, to a substitution rate of $7.9\times 10^{-10}$ substitutions per base pair per year. That is similar to the estimated mitochondrial substitution rate (Table 1).

**Table 1:** Observed fixed and heteroplasmic SNPs at neutral sites in 163 *Z. marina* samples

	Mitochondrial genome	Plastid genome
Total genome length (bp)	187,048	143,968
Average genetic distance between the Atlantic and the Alaskan populations^a^	56.7±8.9	22.5±1.1
Neutral positions for fixed mutations (per sample)	121,502	58,721
Average number of accumulated mutations since the Atlantic–Alaskan divergence (per base pair)^a^	$2.3\times 10^{-4}\pm 3.7\times 10^{-5}$	$1.9\times 10^{-4}\pm 9.4\times 10^{-6}$
Substitution rate (per base pair per year)	$9.6\times 10^{-10}\pm 1.5\times 10^{-10}$	$7.9\times 10^{-10}\pm 3.9\times 10^{-11}$
Substitution rate based on the Californian–Pacific divergence (per base pair per year)	$7.1\times 10^{-10}\pm 8.2\times 10^{-11}$	$7.2\times 10^{-10}\pm 3.7\times 10^{-11}$
Number of heteroplasmic sites^a^	1(+1)	6
Neutral positions for heteroplasmic sites (total^b^)	12,920,202	3,284,422
Number of heteroplasmic sites per base pair (total^b^)^a^	$7.7\times 10^{-8\;}$ ^c^	$1.8\times 10^{-6}$
Heteroplasmic sites^d^	**27,884** | JS03, AF = 0.91 (+124,388 | FR06, AF = 0.51)	SSC: **8,108** | ALI10, AF = 0.75; **11,673** | SD08, AF = 0.44; SD03, AF = 0.48; SD01, AF = 0.82; SD07 and SD11 fixed LSC: **83,431** | ALI10, AF = 0.78; **98,817** | ALI05, AF = 0.50; **118,850** | ALI04, AF = 0.94; ALI05, AF = 0.50; **120,428** | SD08, AF = 0.56

Values are means ± standard deviation. The total number of heteroplasmic sites per base pair corresponds to the expected heteroplasmy probability (*P*) which is the numeric result of simulation experiments. SSC and LSC abbreviations stay for the small single-copy region and the large single-copy region of the plastid genome correspondingly.

^a^At the corresponding neutral positions.

^b^For all the samples in this study together.

^c^For one heteroplasmic site.

^d^Location abbreviations: JS, Japan-South; ALI, Alaska–Izembek; SD, San Diego, California.

An alternative calculation of the substitution rates based on the deepest *Z. marina* divergence between the Californian and the Main Pacific populations yields a similar substitution rate for the ptDNA but a markedly lower substitution rate for the mtDNA (Table 1). Unlike the Atlantic and Alaskan populations that are geographically disjointed, gene flow between the Californian and the Main Pacific populations is likely and was shown for the nuclear genome (Yu et al. 2023). Genetic distances based on the California-to-Main Pacific split might indeed underestimate the substitution rate for mtDNA due to effects such as paternal leakage for the *Z. marina* mitochondrial genome. On the other hand, the clear separation of the Californian haplotypes from the Main Pacific haplotypes points toward a preferential maternal mitochondria inheritance, like for plastids, rather than biparental inheritance as for the nuclear genome (supplementary figs. S1 and S2, Supplementary Material online; Yu et al. 2023). Therefore, to avoid a possible bias caused by paternal leakage, we based our estimations only on the divergence between the Alaskan and the Atlantic populations instead of the deepest divergence between the Californian and the Main Pacific populations.

**Fig. 2.**
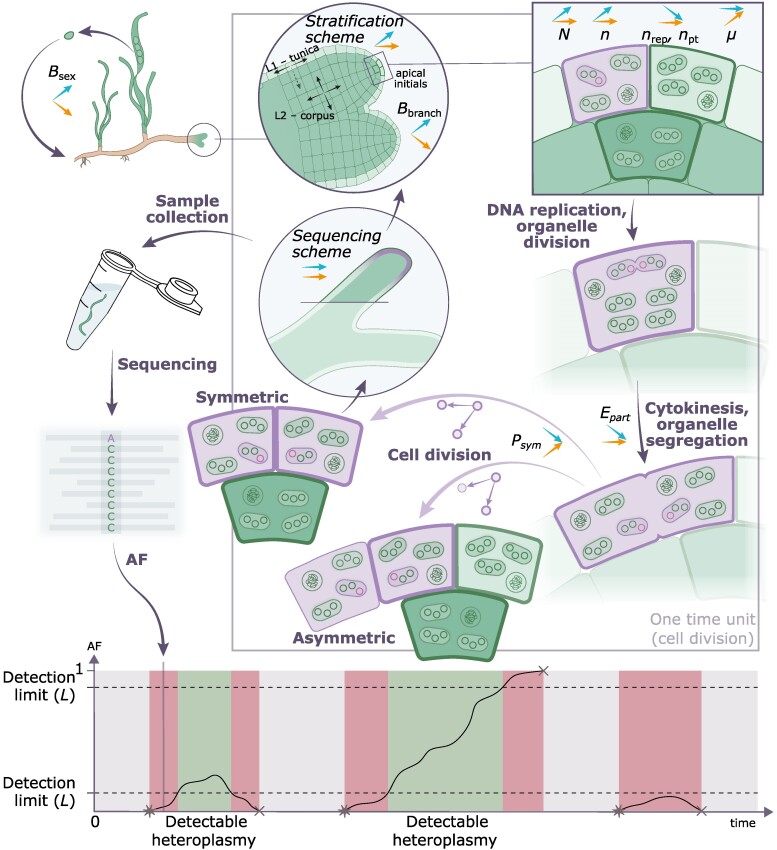
A model for organelle genome evolution within monocot stem cells. The depicted process represents a single stem cell division and highlights the steps and model parameters that affect the composition of the novel (purple) and wild-type (green) alleles in the oDNA population and the AFs detected by sequencing. The cells and organelles (oval shapes) are colored in accordance with their allele composition; assuming the novel allele is dominant, the nucleus (circular shapes) remains unaltered. The parameters and their descriptions are listed in Table 2. Each parameter is accompanied by two arrows that specify the expected effect of its elevation on substitution rate (bottom/orange) and an average neutral allele segregation time (top/blue). The model assumes regular bottlenecks that correspond to sexual reproduction at the *B*_sex_ rate and branching at *B*_branch_ rate. The rest of the model parameters define a single stem cell division process, which is considered one time unit. The graph at the bottom shows the fate of a single locus over multiple time units (stem cell division cycles), where de novo mutations arise (stars) and become fixed or are lost (crosses). The model implies that after every cycle, the meristematic tissue is sequenced, revealing the mutant AF in the focal organellar locus that is either detectable (green area on the graph) or undetectable (red area on the graph) given the limitations of the methodology (*L*). The gray area in the graph corresponds to time units where the locus is in homoplasmic state.

The empirical assessment of mtDNA and ptDNA substitution rates enables us to further compare their mutation rates—the rate at which novel mutations emerge in an oDNA population. Conceptually, the higher copy number of ptDNA compared with mtDNA (Kumar et al. 2014; Greiner et al. 2020) entails an increase in the mutational supply but a decrease in the probability for neutral allele fixation in a manner that is proportional to the replicon copy number *n* (i.e. the copy number should have an effect akin to population size; reviewed in Lanfear et al. 2014). Therefore, we do not expect a positive association between the replicon copy number and substitution rates. In models of single locus dynamics over infinite time (such as the one reported here), genetic drift does not affect the fixation probability of neutral alleles. That being said, population bottlenecks may still have an effect on allele fixation probabilities. Mutations arising just after a bottleneck event (in a reduced cell population) likely have a higher probability of being fixed, since such mutations arise with a higher AF compared with mutations arising in a larger population. Thus, factors affecting the substitution rate include the mutation rate, the proportion of symmetric cell divisions, and the frequency of bottlenecks. The latter two factors manifest at the cellular level and are, therefore, common to plastids and mitochondria. We conclude that *Z. marina* mitochondrial and plastid genomes have similar substitution rates and, thereby, also similar mutation rates. However, estimating the mutation rate from the substitution rate requires modeling of the evolutionary process since multiple factors, such as the proportion of symmetric cell divisions and the frequency of branching and sexual reproduction, affect the fixation probability (Fig. 2).

### Establishment of a Mathematical Model to Investigate the Determinants of Organelle Allele Dynamics

To further examine the determinants of organelle evolution, we established a model to simulate neutral allele dynamics in *Z. marina*. The model describes allele dynamics at a single genome locus until a certain number of mutations occur at this position in the genome and reach either loss or fixation in the oDNA population; the time to loss or fixation is termed here “segregation time.” The ultimate goal of a single simulation run is to calculate the probability (*P*) of detecting heteroplasmy in a focal locus when sequencing plant meristematic region at a random time point (Fig. 2). Later, we adjusted the simulation parameters in order to match the simulation results with our empirical findings. The probability *P* is calculated as the proportion of the time span (in cell divisions) in which the heteroplasmy is detectable (*T*_DET_) to the total time of the simulation (*T*_Total_; Fig. 2). The heteroplasmy was considered detectable if the reference and variant AFs were both above a certain frequency threshold *L*, which reflects the sensitivity of the variant detection methodology. Here, we assume that the considered neutral genome loci are independent and the de novo mutations occur only during the homoplasmic oDNA population state; this assumption is supported by the low mutation rates observed here. Additionally, we assume that the tissue sample is formed of one stem cell descendant and, thus, has the same frequency of the mutant allele as the stem cell itself regardless of the mutant copy distribution among cells (similar to recently published simulations by Broz et al. 2023). We also assume no de novo mutations during tissue formation since the probability for such mutations to arise and reach the detectable AF is low. Thus, the two major components that contribute to the probability *P* are the mutational supply and the average detectable allele segregation time *T*_DET_.

In our model, we consider a set of meristem stem cells to have a constant size of 20 cells organized as a cell bulk with two layers—the tunica layer (L1) and the corpus layer (L2)—each consisting of two rows of five cells. The oDNA copy numbers in stem cells were set according to our estimates of the average mtDNA and ptDNA abundance across 24 samples of the *Z. marina* meristematic region (supplementary table S1, Supplementary Material online). Based on those estimates, we assume that each stem cell contains 40 copies of mtDNA and 216 copies of ptDNA (Table 2; model parameter *n*). Note that previous studies point toward a slightly lower ptDNA copy number, e.g. 35 to 192 copies per cell in beet, *Arabidopsis thaliana*, tobacco, and maize, and slightly higher mtDNA copy number, e.g. up to 65 copies per cell in maize (Kumar et al. 2014; Greiner et al. 2020). Additionally, we introduced the intracellular plastid hierarchical structure by defining the number of ptDNA copies per plastid and plastids per cell individually (model parameters *n*_rep_ and *n*_pt_). Time units in the model correspond to single cell divisions in the population of stem cells (Fig. 2; see model parameters in Table 2). Cell divisions are accompanied by several stochastic processes: (i) the random sampling of the next cell to divide (with equal probabilities for all stem cells), (ii) the choice between symmetric and asymmetric cell division (model parameter *P*_sym_), (iii) the choice between the two daughter cells in case of an asymmetric cell division, (iv) the random sampling of the cell to be removed from the cell population in case of a symmetric cell division (the probabilities are defined by the model parameter *stratification scheme*), and (v) the segregation of replicated oDNA copies to the two daughter cells (that can be random, or defined by the parameter *E*_part_, but is always equal in number between the daughter cells). Additionally, we applied regular bottlenecks of one corpus cell to mimic sexual reproduction events (Alvarez-Buylla et al. 2010) and of ten cells—two vertically stacked rows, one from tunica and one from corpus—to mimic branching events. The frequency of the two bottleneck types is defined by the model parameters *B*_sex_ and *B*_branch_, respectively (Fig. 2 and Table 2). Note that the frequency of branching bottlenecks (*B*_branch_) differs from the frequency of branching events as only one of the two shoot axes undergoes subsampling of stem cells due to lateral bifurcation (as recently shown for *Z. marina*; Yu et al. 2024). After a bottleneck, the cell population is assumed to proliferate until the given constant cell population size is reached.

**Table 2:** Parameters of the agent-based model

Parameter	Definition	Value		Value source	Reference
		mt	pt		
*n*	Number of replicons per cell	40 [up to 65 in maize]	216 [35 to 192]	*Z. marina* Finnish clone sequencing, average among 24 samples	Kumar et al. (2014); Greiner et al. (2020)
*N*	Number of stem cells	20 10 (tunica), 10 (corpus) [2 to 12]		Estimations for *Z. marina* tunica layer	Conway and Drinnan (2017); Yu et al. (2024)
*μ*	Mutation rate (per base pair per replicon per cell division)	$2.6\;\;\times \;\;10^{-11}$ to $5.5\;\;\times \;\;10^{-11}$		Fitting the observed number of accumulated mutations	—
*G*	Number of stem cell divisions per cell per year	40 [34 to 50]		Taken from *A. thaliana*	Lynch(2010); Watson et al. (2016); Burian (2021)
*B*_sex_	Time between sexual reproduction	3 years (strict) 1,000 years (clone) 100 years (relaxed)		General *Z. marina* observations	Yu et al. (2020); Yu et al. (2023)
*B*_branch_	Time between branching bottlenecks	One-fourth year (strict) One-fourth year (clone) 1 year (relaxed)		General *Z. marina* observations	Harrison (1993); Greve et al. (2005)
*P*_sym_	Probability for a cell division to be symmetric in a constant size cell population	0.01		Much lower than 0.5, no exact calculations	Pineda-Krch and Lehtilä (2002); Harrison et al. (2007); Burian (2021)
Stratification scheme^a^	Which cells are replaced during a symmetric cell division, and their replacement probability	Tunica layer anticlinal to periclinal cell divisions in proportion 10:1		Periclinal divisions are rare, no exact calculations	Pineda-Krch and Lehtilä (2002); Burian (2021)
*n*_pt_	Number of plastids per cell	—	12 [7 to 11]	*Z. marina* Finnish clone sequencing, average among 24 samples	Sheahan et al. (2007); Greiner et al. (2020)
*n*_rep_	Number of ptDNA copies per plastid	—	18 [8 to 24]	*Z. marina* Finnish clone sequencing, average among 24 samples	Greiner et al. (2020)
*E*_part_	Active partitioning error	0.5	0.00058 to 0.00073	Fitting the observed number of heteroplasmic sites	—
*L*	Detection limit	5%		Variant calling limitations	Fang et al. (2015)
*M*_fix_	Expected number of fixed mutations during a simulation experiment	200, 500, 2,000		Simulation convergence	—
Sequencing scheme^a^	The fractions of stem cell progenies in a collected tissue sample	All equal		Simulation shows little difference when changing this parameter	—

The values used as default in the simulations for mitochondria (mt) and plastids (pt) were taken from literature and *Z. marina* experiments when possible. Numbers in square brackets show the value ranges based on existing data.

^a^The parameter is nonnumerical per se, yet its effect on the simulation results is quantified numerically.

### Organelle Mutation Rates as Inferred by the Model

**Fig. 3.**
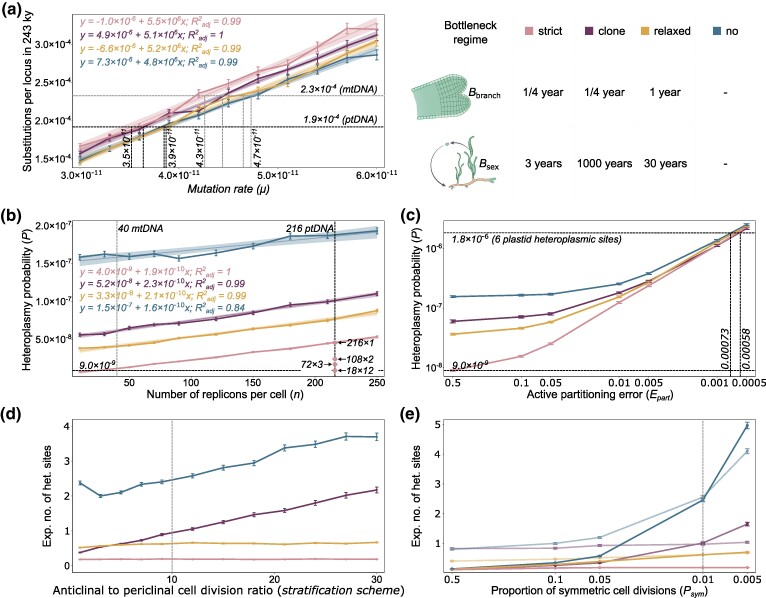
The effect of individual parameters in the simulation of *Z. marina* evolution of organelle genomes in the absence of selection. a) The number of mitochondrial substitutions per locus in 243,300 years in the simulation experiments for different mutation rates (*µ*). The vertical-dashed gray (mitochondrial genome) and black (plastid genome) lines indicate the *µ* values matching the observed number of accumulated fixed mutations (horizontal-dashed lines). The legend shows the colors and the parameters used for the four bottleneck regimes in Figs. 3 and 4. b) The alteration of the heteroplasmy probability (*P*) with an increase in the replicon copy number (*n*) for *µ* that corresponds to the plastid genome. Points show the estimated *P* when applying the plastid hierarchical structure in the range of 216 ptDNA/pt for 1 pt/cell to 18 ptDNA/pt for 12 pt/cell. Horizontal black-dashed line indicates the *P* values for ptDNA with the strongest hierarchy. Vertical-dashed lines indicate the default copy number values for mtDNA (gray) and ptDNA (black). c) The estimated probability *P* when an active plastid segregation is applied with a certain active partitioning error (*E*_part_). Horizontal-dashed gray lines indicate the *P* value for a random partition (in the bottom) and the expected *P* based on the observation data (in the top). Vertical-dashed lines indicate the predicted *E*_part_ range based on the observation data. d) The dependency of the expected number of mitochondrial heteroplasmic sites in the worldwide data set when increasing the tunica and corpus layer isolation. The vertical-dashed line indicates the default stratification parameter. e) The effect of the symmetric cell division proportion (*P*_sym_) with the fixed level of stratification (bold lines) and adjusted stratification level (transparent lines) on the expected number of mitochondrial heteroplasmic sites. The vertical-dashed line indicates the default *P*_sym_ parameter.

In order to estimate the oDNA mutation rates (*μ*), we simulated the allele dynamics for different *μ* values to calculate the expected number of substitutions within the oDNA population in a single locus in 243,300 years. This result, multiplied by the number of sites, thereby corresponds to the expected number of accumulated mutations in a *Z. marina* individual since the Alaskan–Atlantic divergence. Under a neutral regime, the mutation rate equals the substitution rate (Kimura 1968). Indeed, we found the substitution rate and the mutation rate *μ* to be linearly dependent, enabling us to estimate the mutation rate (*μ*) from the value that best fits the observed number of fixed mitochondrial and plastid mutations (Fig. 3a). Bottleneck regimes with high branching rates—“strict” and “clone”—show a similar trend, and so are bottleneck regimes with low branching rates—“relaxed” and “no”; hence, the frequency of branching had a major effect on the estimated *μ* when applying different bottleneck regimes. However, the total range of the estimated mutation rate remained within a narrow range of $4.3\times 10^{-11}\;$ to $4.7\times 10^{-11}$ and $3.5\times 10^{-11}$ to $3.9\times 10^{-11}$ mutations per base pair per replicon per cell division for mtDNA and ptDNA, respectively (Fig. 3a). Likewise, the proportion of symmetric cell divisions (*P*_sym_) had a minimal effect on the estimated mutation rate (*μ*) if considered within a realistic range 0.1 to 0.005 (supplementary fig. S3, Supplementary Material online). To transform the unit of time (total number of cell divisions) used in our model into time elapsed in years, we used a constant cell depth, *G* = 40 cell divisions per stem cell per year, as for an annual plant *A. thaliana*. Although eelgrass might refrain from sexual reproduction for tens of years, eelgrass ramets are susceptible to selection within a single clone. Hence, we assume that the number of stem cell divisions per year in eelgrass is similar to those of annual plants. The mutation rate is proportional to the cell depth, *G*, which is unlikely a value outside the range of 34 to 50 cell divisions per stem cell per year (reviewed in Burian 2021). Applying this cell depth range to our estimates, we infer that the absolute mutation rate of *Z. marina* mitochondria is between $3.2\times 10^{-11}$ and $5.5\times 10^{-11}$, and in plastids, it is between $2.6\times 10^{-11}$ and $4.7\times 10^{-11}$ mutations per base pair per replicon per cell division (note that a rough estimate of the mutation rate can be achieved also by dividing the substitution rate by *G*).

### Allele Segregation Is Approximately 20-Fold Slower in Plastids Compared with Mitochondria

To further compare the neutral allele segregation times between mitochondria and plastid genomes, we examined the number of heteroplasmic loci that may reveal ongoing allele segregation across multiple sequenced samples. For that purpose, we identified all heteroplasmic SNPs at neutral positions for heteroplasmic sites across all 163 available *Z. marina* samples collected in 16 locations of the Pacific and Atlantic Oceans (Table 1). In total, one heteroplasmic locus was detected in mitochondrial genomes with one additional heteroplasmy of an unclear origin. Six heteroplasmic loci were detected in the plastid genomes of the 163 samples. Since the total number of neutral positions for heteroplasmic sites is approximately four times higher for mitochondria compared with plastids (Table 1), we conclude that heteroplasmy is approximately 20 times more abundant in the plastid compared with the mitochondrial genome.

As a next step, we used our model to identify the factors that would be able to explain such a dramatic difference in observed heteroplasmy. We focused on three factors that distinguish mitochondrial and plastid allele segregation dynamics: replicon copy number, hierarchical structure, and mode of segregation (i.e. partition mechanism). The simulation demonstrates that when the replicon copy number increases from 40 (*n*_mtDNA_) to 216 (*n*_ptDNA_), *P* increases 1.2- to 3.9-fold depending on the bottleneck regime (Fig. 3b), which is much lower than the observed 20-fold difference in the number of heteroplasmic sites per base pair (as observed in the empirical data). Notably, the simulation results show a linear association between heteroplasmy probability and the copy number. The linear association is well explained by the constant total number of cell divisions in a single simulation experiment and the average time of detectable heteroplasmy (*T*_DET_) that is nearly constant in the simulation, such that the increase in copy number affects solely the mutational supply, resulting in a linear dependency (supplementary fig. S4a and b, Supplementary Material online). The observation of nearly constant detectable segregation time implies that the increased segregation time is being buffered by the increased number of mutant replicons that allow the detection of heteroplasmy (i.e. depending on the detection limit threshold *L*). Furthermore, intracellular hierarchy which is distinctive for plastids significantly decreases the segregation time, which results in a heteroplasmy probability for ptDNA that is even slightly lower than for mtDNA (Fig. 3b, see point for 18 × 12; supplementary fig. S4c, Supplementary Material online). Thus, we reasoned that the third factor—the mode of segregation—is the only one remaining to explain the difference in the number of observed heteroplasmic sites. Here, we assume that nuclear control of equal plastid segregation to different daughter cells in stem cells occurs via an active partition of sister plastids after plastid division. In an extreme scenario, such a mode of segregation would never lead to allele fixation in the absence of biparental inheritance, as shown in the example of nuclear mutations in clones (Yu et al. 2020). However, the fixation of novel alleles is possible if we allow a certain probability of partitioning error; we use parameter *E*_part_ in our model to define the partitioning error. A partitioning error of 0.5 indicates that two sister plastids segregate to the same daughter cell with a probability of 0.5, thus simulating random segregation. The simulation demonstrates that a partitioning error of 0.00058 to 0.00073 results in a heteroplasmy probability *P* that is consistent with the observed number of ptDNA heteroplasmic sites in the sequencing data (Fig. 3c). Thus, we conclude that the observed difference between plastid and mitochondrial allele dynamics is likely explained by a relatively strict active partition of plastids during stem cell division, very much in contrast to the random segregation of mitochondria. Notably, when applying strict active partition, intraplastid homoplasmy is reached considerably fast (within 740 to 1,140 cell divisions, on average, depending on the bottleneck regime, which corresponds to 0.9 to 1.4 years), while the heteroplasmy is maintained in the ptDNA populations by active partition among plastids in cells, as cells reach homoplasmy 300 to 550 times slower. Consequently, the number of plastids per cell has a minimal effect on the segregation time, if the number of ptDNA copies per plastid remains constant (supplementary fig. S4e, Supplementary Material online). In contrast, when applying random plastid segregation (*E*_part_ = 0.5), the average time until the first homoplasmic plastid occurs (560 to 740 cell divisions, which corresponds to 0.7 to 0.9 years) is similar to the time until the first homoplasmic cell occurs (1,200 to 1,700 cell divisions, which corresponds to 1.5 to 2.4 years).

### Meristem Stratification Increases Allele Segregation Time in the Absence of Frequent Sexual Reproduction

In the next step, we performed simulations to evaluate the effect on organellar allele dynamics of four parameters that are acting on the cellular level and, therefore, are common for both organelles: the *stratification scheme*, the probability of symmetric cell division (*P*_sym_), the *sequencing scheme*, and the number of stem cells (*N*). To evaluate the effect of the *stratification scheme* in the model, we applied different ratios of the anticlinal to periclinal cell division in the tunica layer, which reflects stratification between the tunica (L1) and the corpus (L2) meristematic layers. Periclinal divisions occur only during the nonproliferating stage and only in the direction from tunica to corpus in our model. The simulation results predict a strong impact of the frequency of sexual reproduction (*B*_sex_) on the effect of stratification since the regular single-cell bottlenecks inevitably admix the genetic content of the two meristematic layers such that the effect of stratification is limited (Fig. 3d, bottleneck regimes “strict” and “relaxed”). For the bottleneck regimes featuring a clonal lifestyle, however, the relatively strict genetic isolation of the two layers leads to an increase in the expected number of heteroplasmic sites (Fig. 3d, bottleneck regimes “clone” and “no”). Decreasing the parameter *P*_sym_, proportion of symmetric cell divisions, significantly increases the probability *P* and thereby the expected number of heteroplasmic sites (Fig. 3e). However, the major contribution to the observed elevation arises from the corresponding decrease of the periclinal genetic material flow from the tunica to the corpus. The individual effect of the parameter *P*_sym_ is minimal and buffered by branching bottlenecks that inevitably imply that cells are replaced during the proliferation stage (see the effect of stratification in Fig. 3e). Furthermore, we considered one factor of the system that does not affect the segregation dynamics but might alternate the estimated AF—the proportion of the stem cell progenies in the sequenced tissue, *sequencing scheme*. The sequenced meristematic region contained early-developed tissues, such as leaf primordia. Since the periclinal division is common in leaf formation, the proportion of tunica and corpus progenies is difficult to estimate. We conducted the calculations assuming the proportion of the tunica layer progenies to the corpus layer progenies from 16 to 1/16 and found only small differences in the estimated expected numbers of heteroplasmy sites between the bottleneck regimes with bottlenecks. Hence, we concluded that disproportional contribution of the stem cells to the sequenced tissue has a minimal effect (supplementary fig. S4d, Supplementary Material online). Therefore, we used an equal number of progenies for all stem cells by default in our simulations. Similarly, the number of stem cells (*N*) has a minimal effect on both the allele fixation and the allele segregation processes when bottlenecks are applied (supplementary fig. S5, Supplementary Material online).

If we use an extreme combination of the simulation parameters *stratification scheme* of 30:1 (anticlinal to periclinal cell divisions) and $P_{\mathrm{sym}}=0.005$ and ignore bottlenecks, the expected number of heteroplasmic sites in 163 samples is 7.8 for mitochondria and 1.7 for plastids. Notably, under these extreme parameters, the estimated number of heteroplasmic sites for the mitochondria is considerably higher than the observed one (or two) sites in the sequencing data, while the estimation for plastids remains significantly lower than the observed six heteroplasmic sites. Note that we considered stochastic partition, *E*_part_ = 0.5. Hence, our analyses of the effects of parameters support our hypothesis that the partition of sister plasmids to the daughter cell is not random.

### Plastids but Not Mitochondria Are Expected to Have Common Heteroplasmic Sites between Different Clones of the Same Eelgrass Population

**Fig. 4.**
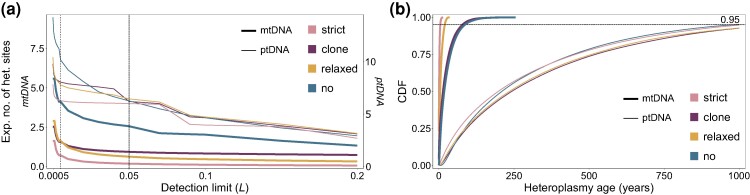
Allele dynamics in a single simulation experiment of *Z. marina* mtDNA and ptDNA neutral evolution. a) The expected number of heteroplasmic sites in 163 samples in mitochondrial (bold lines) and plastid (thin lines) genomes for different heteroplasmy AF detection limits (*L*). The vertical-dashed line indicates the default detection limit of 0.05; the vertical-dotted line indicates the detection limit of 0.005. b) The cumulative age distribution of detectable heteroplasmy in mitochondria (bold lines) and plastids (thin lines). Colors correspond to the bottleneck regimes in accordance with Fig. 3.

To evaluate the limitation of the whole-genome sequencing approach for detecting heteroplasmy, we applied different detection limits *L*. Note that the total number of ptDNA copies in the focal oDNA population in the simulation is 4,320 (216copiespercell×20cells) and the total number of mtDNA is 800 (40copiespercell×20cells). A threshold of zero features an accurate allele detection process with one mutant copy to be sufficient for heteroplasmy detection, while the highest threshold of 0.2 requires at least 864 plastid and 160 mitochondrial mutant copies for the detection. Our results demonstrate that a 10-fold decrease in *L* from the default value of 0.05 to 0.005 only shows a slight increase in the expected number of heteroplasmic sites for both mitochondria and plastids (Fig. 4a). Yet, the spike for *L* = 0 shows the maximum number of heteroplasmic sites in 163 samples to be 5.6 for mtDNA and 14.2 ptDNA. Thus, the number of novel neutral mutations in a heteroplasmic state in a single sampled eelgrass individual is expected to be below one while heteroplasmy can be expected for a large number of samples. Consequently, we conclude that if an organelle heteroplasmy is detected in a single sequenced individual, it is unlikely to refer to an ongoing segregation of a novel mutation.

To this end, we used the selected simulation parameters that are in accordance with plant biology and the observed data to estimate the age distribution of detectable heteroplasmic sites in mitochondria and plastids. The simulation indicates that while 67% to 73% of the detectable heteroplasmic sites in plastids are over 100 years old and 5% to 7% are over 1,000 years old, only 2% to 3% of mitochondrial heteroplasmic sites persist for at least 100 years given a clonal lifestyle (Fig. 4b). Eelgrass populations with regular sexual reproduction are not expected to maintain intraindividual mitochondrial heteroplasmy for over 36 years (Fig. 4b). Our prediction that old heteroplasmy can be found in the plastid genome is confirmed by the empirical data. Two out of six detected heteroplasmic sites in the plastid genome—position 11,673 in the San Diego population and position 118,850 in the Alaska–Izembek population—are heteroplasmic at the level of individual plants. At the same time, these two sites have a fixed mutant genotype in five (position 11,673) and two (position 118,850) samples taken from the same location (Table 1). Given that all the samples belong to different clones, the mutations likely emerged in the ancestor of the sampled clones, thereby persisting in the eelgrass population for a significant time and surviving at least one single-cell bottleneck. The presence of heteroplasmy in multiple clones in the plastid genome but not in the mitochondrial genome might be an additional indirect confirmation for our conclusion that the segregation time of plastid alleles is significantly elongated.

## Discussion

Studying the evolution of organelles is crucial for our understanding of plant adaptation to changing environments due to their contribution to cellular respiration, photosynthesis, and a myriad of other essential processes. Here, we show how the organization and partition of the plant organelles within the germline affect their genome evolution. In doing so, we uncover the fundamental principles of organelle population genetics that are essential for further investigations of long-term evolution and molecular dating of divergence events.

Mutation rates are generally estimated for nuclear genomes and in units per plant generation (Krasovec et al. 2018; Zhang et al. 2018). More rarely, the mutation rate is given per stem cell division, which allows comparison across species; this rate might be as low as $1\times 10^{-11}$ in lotus (*Nelumbo* Adans.) in comparison with $1.6\times 10^{-10}$ in *A. thaliana* (Lynch 2010; Ossowski et al. 2010; Zheng et al. 2022). Since organelle genomes are much smaller than the nuclear genome (usually <400 kb), estimates of evolutionary rates, for example, synonymous substitution rates or neutral substitution rates, based on mutation accumulation require observations over longtime scales. The majority of estimations for the plant organelle evolutionary rates are based on synonymous and nonsynonymous substitutions in cross-species comparisons (Wolfe et al. 1987; Muse 2000; Drouin et al. 2008; Xu et al. 2012). These studies consistently show a significantly faster plastid gene evolution in comparison with mitochondrial gene evolution (Wolfe et al. 1987; Drouin et al. 2008). Individual analysis of plastid genomes showed that inverted repeats evolve slower compared with single-copy loci, similar to the rates for mitochondrial genomes (Muse 2000). However, only a few studies measured the evolutionary rates of mitochondrial and plastid genomes for individual species. One example is a previous study of Douglas fir (*Pseudotsuga menziesii*) that shows a similarity between the two organelle neutral substitution rates of $5.26\times 10^{-10}$ and $4.41\times 10^{-10}$ substitutions per base pair per year for mtDNA and ptDNA, respectively (Gugger et al. 2010), which are slightly lower compared with eelgrass (Table 1). The similarity between mitochondrial and plastid substitution rates is concordant with the similarity in their core enzymes and components of the oDNA replication system, which are identical for plastids and mitochondria in land plants (Moriyama and Sato 2014). Why studies based on interspecies comparison result in significantly different mutation rates between the two land plant organelles, while the single-species studies demonstrate the similarity of mutation rates, remains a conundrum. One possible explanation is the long-term effect of DNA recombination, which may be strong for mtDNA and inverted repeats in ptDNA, but not as strong for ptDNA single-copy loci (reviewed in Maréchal and Brisson 2010). In the absence of frequent recombination, ptDNA single-copy regions might exhibit a high frequency of neutral mutations due to linkage with regions evolving under positive selection, which increases the estimated neutral substitution rate. Recombination is likely to break linkage disequilibrium between beneficial and neutral mutations, thus hindering the fixation of passenger mutation. Notably, all observed plastid heteroplasmic sites were located in single-copy regions (Table 1). Hence, the neutral allele segregation time in the inverted repeats may be shorter than in the single-copy regions. The observed difference might be a direct consequence of the frequent recombination of the inverted repeats as well.

Both the observed number and the model prediction of heteroplasmic sites are much lower than the number of samples even if all variants are detected, i.e. the detection limit has no effect. This suggests that detecting any heteroplasmy in a single sequencing experiment is unlikely. To find sufficient heteroplasmic sites in organelles, one would need to sequence multiple samples from multiple populations. Our conclusion agrees with Scarcelli et al. (2016), who suggested that plastid heteroplasmic sites in sequencing data likely stem from shared DNA fragments among plastids, mitochondria, and the nucleus (i.e. NUMTs, NUPTs, and mtptDNA) that quickly accumulate mutations after transfer (Michalovova et al. 2013; Sloan and Wu 2014). In addition to intraindividual polymorphisms created by shared DNA, we observed that mitochondrial small imperfect repeats may generate false-positive heteroplasmy signals. At the same time, considering one additional mtDNA heteroplasmic position to be genuine (Table 1) would not change the main conclusion we draw from the sequencing data. The difference between plastid and mitochondrial heteroplasmy abundance would be thereby 12-fold, which we explain by a difference in the organelle partition mechanism. Moreover, since the plastid heteroplasmy is expected to be observed in closely related clones (see Fig. 4b), our assumption that the sequenced samples are independent may lead to an overestimation of the expected number of heteroplasmic sites from the heteroplasmy probability *P*. The expected number of heteroplasmic sites is likely to be overestimated in mitochondria as well due to regular mtDNA recombination and possibly also relaxed replication (Chinnery and Samuels 1999) that were not considered in our simulations. Both factors are expected to either eliminate rare novel alleles or accelerate allele segregation through their contribution to the homogenization of allele diversity within the cell. Since our results indicate that the propagation of mitochondrial mutations in the cell population is a key determinant of allele dynamics, factors accelerating allele fixation within cells are expected to have a small effect on our results. Furthermore, our recent report of a high conservation of mitochondrial genome architecture in *Z. marina* suggests that the frequency of relaxed replication in meristematic cells is low (Khachaturyan et al. 2023). Notably, a study of different date palm cultivars shows a similar pattern, where all detected mitochondrial heteroplasmic sites were unique for specific cultivars, while all of the detected plastid sites were heteroplasmic in all of the cultivars (Sabir et al. 2014). This observation suggests that the dramatic difference in the allele segregation time for mitochondria and plastids might not be a unique characteristic of eelgrass but rather a common evolutionary principle across plant taxa.

Our simulation proposes an active partition of plastids during cell division. To our knowledge, the fate of sister plastids following stem cell division has not been shown experimentally in any plant species. However, evidence has been presented for the participation of actin filaments and for the codependence of the cell and plastid cycles, suggesting a nonstochastic partition (Sheahan et al. 2004; Chen et al. 2009; reviewed in Pedroza-Garcia et al. 2016). The nuclear control over plastid division is stronger in meristematic cells (Swid et al. 2018). In monoplastidic organisms, such as basal-branching algae, the plastid and nuclei division and the partition during the cytokinesis are well coordinated (Sumiya et al. 2016). More recently, it has been suggested that the evolution of a nuclear control of the plastid cycle in land plants enabled the switch from monoplastidity to polyplastidity (de Vries and Gould 2018). Yet, the mechanisms of plastid copy number control in stem cells are likely to remain unchanged. Consequently, the relocation of plastids from the perinuclear area to the daughter newly formed nuclei is likely to be achieved by active pulling of sister plastids to the opposite poles. Although active partition is understudied for eukaryotic ECEs, mechanisms of active partition are widely described for plasmid segregation in bacteria (reviewed in Bouet et al. 2007 and Baxter and Funnell 2015).

In our simulations, we estimated the individual effects of multiple parameters on the dynamics of allele segregation. The fixation time in mtDNA populations depends primarily on the rate of the mutation propagation to other cells and meristematic layers. Therefore, the stratification significantly extends the mitochondrial allele segregation time given that the population undergoes few single-cell bottlenecks (i.e. with rare sexual reproduction events). This result is in agreement with studies showing that stratification and cell-to-cell competition decrease the accumulation of deleterious mutations (Pineda-Krch and Lehtilä 2002; Klekowski 2003; Edwards et al. 2021). A higher number of stem cells do not result in an increased segregation time. Thus, the major drivers of the mutation propagation within a meristematic layer are likely bottleneck events during stem branching and seeding, which are followed by cell proliferation until the total number of stem cells is reconstituted. In contrast, if we indeed assume, as the model suggests, the presence of an active partition mechanism for plastids, the limiting process in the new plastid allele segregation is the within-cell fixation rather than the mutation propagation among cells. In that case, the key parameter leading to the extension of plastid allele segregation time is the plastid partitioning error (*E*_part_). Our model shows that the fixation of new mitochondrial variants is achieved relatively fast at all levels of organization, including individual organelles and cells, separate branches, and individual plants. The presence of mitochondrial genetic diversity at multiple levels implies that purifying selection may act at different organizational levels as well. In contrast, plastid variants are fixed within individual organelles, yet the plastid genome remains heteroplasmic at all higher levels of organization over a long period of time, and that may hinder purifying selection against slightly deleterious plastid alleles. Unlike mitochondria, whose function is relatively uniform across tissues, proplastids in meristematic cells perform only basal metabolic functions. We speculate that potentially deleterious or beneficial plastid mutations may be neutral at the level of meristematic cells. Consequently, the competitive pressure among stem cells might be weaker for slightly deleterious plastid variants than for mitochondrial variants, even if allele fixation is reached at the cellular level. How the presence of organelle heteroplasmy at different levels of organization figures into plant adaptation remains a matter for further investigation.

Notably, the effect of drift and selection at multiple levels of organization has been recognized in other ECEs, e.g. prokaryotic plasmids (Ilhan et al. 2019). Indeed, efforts to model ECE dynamics and evolution typically encounter similar challenges (e.g. Hunter and Fusco 2022; Santer et al. 2022; Broz et al. 2023). Nonetheless, despite common evolutionary characteristics, research on the biology and evolution of oDNA and other ECEs is typically disjoined due to disciplinary boundaries. The development of evolutionary theories and mathematical models of ECE population genetics supplies a basis for cross-disciplinary evolutionary research.

## Materials and Methods

### Data

Chromosome-level nuclear and plastid *Z. marina* genome assembly were downloaded through the ORCAE platform (https://bioinformatics.psb.ugent.be/gdb/zostera/; Ma et al. 2021). The complete mitochondrial genome assembled in Khachaturyan et al. (2023) was obtained from the NCBI (accessions: OR336317 and OR336318). The Illumina whole-genome sequencing of *Z. marina* meristematic region of the inner leaf base was obtained from JGI (proposal ID: 503251) for the worldwide population data set and from the NCBI (BioProject: PRJNA557092) for the Finnish clone at Ängsö data set (Yu et al. 2020, 2023). Worldwide population data set samples were preselected according to Yu et al. (2023) in order to avoid an effect of selfing on the detected variants and to ensure that no two samples belong to the same clone (i.e. all samples are separated by at least one sexual reproduction event; Yu et al. 2023).

### Detection of Genetic Variants

We used the *Z. marina* whole-genome sequencing data set that comprises 163 samples of meristematic region tissue collected in 16 locations of the Atlantic and Pacific Oceans. High-quality (88% of base pairs have the Phred quality score ≥ 30) raw Illumina short reads of the 163 samples were aligned to the reference plastid and mitochondrial genomes by BWA-MEM with default parameters and processed by SAMtools v1.10. The median coverage of the mtDNA regions included in this study ranged between 5× and 5,284× with an average median of 985 for all samples. The median coverage of ptDNA regions included in this study was between 395× and 20,472× with an average median of 6,966×. AFs were calculated by pysamstats (Li 2013; Danecek et al. 2021; https://github.com/alimanfoo/pysamstats). Additionally, variants were called by BCFtools mpileup v1.8 with unlimited alignment depth, minimum base quality -Q 20, and the --count-orphans parameter (Danecek et al. 2021). DNA regions shared between mitochondria and nucleus (NUMTs), or plastids and nucleus (NUPTs), or mitochondria and plastids (mtptDNA) were identified with BLAST (Altschul et al. 1990) of the reference mitochondrial and plastid genomes against the nuclear genome and against each other with -evalue 10^−4^ -word_size 11 -reward 2 -penalty -3 -gapopen 5 -gapextend 2 -perc_identity 70 parameters previously used by Smith et al. (2011). In order to avoid undetected shared DNA regions, misaligned repeats, or other artifacts of an unknown origin in heteroplasmy detection, we included a measure that we term here “coverage proportion” for heteroplasmy filtration. The measure was calculated as the ratio of the sequencing coverage of individual positions in both organelle genomes to the median coverage of the corresponding genome segment, excluding the shared DNA regions previously detected by BLAST (see also Khachaturyan et al. 2023). Genome segments were defined in accordance with the mitochondrial genome rearrangements for mitochondria and in accordance with the quadripartite structure separating the single-copy and inverted repeat area for plastids.

For the estimation of the substitution rate and heteroplasmy abundance, we first identified neutral positions in both organelle genomes, i.e. positions in which a potential mutation could be classified as a de novo neutral mutation. We refer to such positions later on as “neutral for fixed mutations” or “neutral for heteroplasmic sites.” As the next step, we identified fixed and heteroplasmic variants at the level of an individual plant in the corresponding neutral positions across all 163 samples. To minimize the possibility of missed heteroplasmic sites (i.e. false-negative heteroplasmy), we initially used the raw sequencing read data and a relatively high AF threshold of 0.05. For the identification of heteroplasmic sites, we additionally applied several filtration steps in order to exclude false-positive results; the remaining variants were furthermore inspected manually for possible artifacts (as described below).

Neutral positions for fixed mutations and heteroplasmic sites comprised positions in noncoding and nonmicrosatellite areas. We excluded the coding areas completely since even the synonymous mutations in organellar genomes are not necessarily neutral (Sloan and Taylor 2010). Microsatellites are recognized hotspots for read misalignment and increased mutability. Consequently, positions in, or in the vicinity (≤2 bp), of mononucleotide repeats longer than eight base pairs were signaled as associated with microsatellites and excluded. Neutral positions for fixed mutations in mitochondria were filtered to exclude mtptDNA areas because plant cells are expected to have a higher ptDNA copy number compared with mtDNA. For the identification of neutral positions for heteroplasmic sites, we applied the same criteria as for the fixed mutations with several additional filtration steps. In both organelle genomes, neutral positions for heteroplasmic sites were filtered to exclude all shared DNA regions (i.e. NUMTs, NUPTs, and mtptDNA). In the analysis of mitochondrial positions, we excluded completely 9 samples that had a median coverage below 50. Furthermore, individual positions were excluded if they had a coverage of less than 150 or if they had a coverage proportion less than 0.3 or higher than 1.5. In the analysis of plastid positions, we excluded the inverted repeat (IRa and IRb) loci, as they mutate at a different rate compared with the single-copy regions (Wolfe et al. 1987; Zhu et al. 2016). Furthermore, individual positions with the coverage proportion below 0.3 were excluded. The resulting number of mitochondrial and plastid neutral positions for fixed mutations and heteroplasmic sites is reported in Table 1.

An SNP was considered fixed if the variant AF was ≥0.75 for both organelles. Here, we applied a considerably low AF threshold for mutation fixation in order to account for variants in shared genome regions (see also Yu et al. 2023 Supplementary Fig. 8). The fixed variants were used to infer the substitution rate by estimating the genetic distance (i.e. minimum number of nucleotide substitutions) between pairs of samples. The genetic distance between groups of samples was calculated as the average genetic distance between each pair of samples from the compared groups (Alaskan and Atlantic and Californian and Main Pacific). The substitution rate was estimated as follows:


$$\frac{\mathrm{genetic}\;\mathrm{distance}}{2\times N_{\mathrm{neutral}\;\mathrm{pos}}\times T_{\mathrm{divergence}}}$$

where *N*_neutral pos_ is the number of neutral positions for fixed mutations in a single genome and *T*_divergence_ is the divergence time of the corresponding branches in accordance with Yu et al. (2023).

In both oDNA, an SNP was considered a heteroplasmic site if the AF was between 0.05 and 0.95 based on both the raw read alignment and the BCFtools mpileup output. We additionally excluded mitochondrial heteroplasmic sites that (i) were clustered together, i.e. more than one potential heteroplasmic site were within two nucleotide distance from each other, (ii) had the coverage proportion of the reference allele above one in at least one sample (in this case, the heteroplasmy can be explained by extra coverage of reads that likely originated from another DNA region), and (iii) stemmed from alignments of imperfect repeats. Among the remaining three mitochondrial heteroplasmic sites, the variant in position 74,138 had an AF > 0.99; hence, it was fixed in most Californian samples and is therefore unlikely to be a genuine neutral de novo mutation, which arose in the last common ancestor of these populations. We hypothesize that this variant emerged via biparental inheritance of mitochondria. The variant in position 124,388 with AF 0.51 that was detected in one of the Mediterranean France population (FR06 sample) was suspected due to high similarity in AF to several potential heteroplasmic sites from the same sample and other samples of the same geographic location, which we removed during the filtration step. All those positions had an AF close to 0.5 in one of the Mediterranean France samples and were fixed in all Pacific populations. Hence, that position (124,388) is not considered here a genuine de novo mutation (but is listed in Table 1). From the seven identified plastid heteroplasmic sites, we manually excluded position 119,234 (sample WN10; North Wales population) due to a possible association with a microsatellite of seven repeated adenines. To avoid sequencing errors mimicking natural heteroplasmy, we furthermore used BCFtools mpileup to confirm that all the identified heteroplasmic sites have high-quality forward and reverse reads aligned to the reference and variant nucleotide. All the samples in which we identified heteroplasmy, except for FR06, had an additional resequencing data that confirmed the presence of the detected heteroplasmy. Lastly, for the sample JS03, another sample JS04 from the same clone was collected and sequenced (Yu et al. 2023). The presence of the mitochondrial heteroplasmy in position 27,884 was confirmed also in that sample (JS04), where it had a slightly shifted AF (AF = 0.94). Altogether, we identified one to two neutral heteroplasmic sites in the mitochondrial genome and six neutral heteroplasmic sites in the plastid genome (Table 1).

### Estimation of Replicon Copy Number

To estimate the replicon copy number (*n*) parameter, we analyzed 24 meristematic region samples of *Z. marina* ramets that belong to a single clone (Yu et al. 2020). The short-read data were aligned to the plastid and mitochondrial genomes by BWA-MEM with default parameters, and the coverage was calculated directly by SAMtools. The average coverage was calculated for nonshared organelle genome areas (i.e. excluding NUMTs, NUPTs, and mtptDNA) and further normalized to the target coverage of the sequencing depth per diploid genome taken from Yu et al. (2020) that was 81×. The resulting replicon copy number per cell for the mtDNA varied between 11 and 73 with an average of 40 copies per cell. The copy number of ptDNA ranged between 153 and 316 with an average of 216 copies per cell (supplementary table S1, Supplementary Material online).

### Simulation

Stochastic agent-based computer simulations (see review in Helbing 2012) that implement the mitochondrial and plastid neutral evolution model were performed using Python v3.10.5. The scripts are available at https://github.com/zyukeriya/plantOrganelleEvolSim. For each parameter set, the simulation was run until the number of mutations that arose in the oDNA population reached a threshold of $n\times N\times M_{\mathrm{fix}}$, where *n* is the replicon copy number per cell and *N* is the number of stem cells. The *M*_fix_ parameter is a constant that was implemented in order to equalize the expected number of mutations that will reach fixation in a single simulation run. The value of *M*_fix_ was arbitrarily set to 2,000 for the mutation rate calculation experiments, 500 for the mitochondrial evolution simulations, and 200 for the plastid evolution simulations. This setting enables the simulation results to converge.

The time between mutation events was drawn from a geometric distribution with the success (mutation) probability:


$$p=(1-{\left(1-\mu \right)}^{n})\left(1-F_{\mathrm{asym}}+\frac{F_{\mathrm{asym}}}{2}\right)$$

where the fraction of asymmetric cell divisions is as follows:


$$F_{\mathrm{asym}}=\frac{\left(B_{\mathrm{sex}}-N+1)(B_{\mathrm{branch}}-N/2)(1-P_{\mathrm{sym}}\right)}{B_{\mathrm{sex}}B_{\mathrm{branch}}}$$

*μ*, *n*, *B*_sex_, *B*_branch_, *N*, and *P*_sym_ are defined in Table 2. The estimation error was calculated as the standard error of the mean for the distribution formed by the outcomes of individual mutations. Effects of the *sequencing scheme*, the detection limit (*L*), and the distribution of heteroplasmy age were inferred from a single run of the simulation. In all simulations, we used a range of mutation rate (*μ*) values in combination with different sets of *P*_sym_ values and bottleneck parameters that yielded substitution rate estimates fitting the observed substitution rates (see Fig. 3a). Similarly, for the plastid evolution simulation with an active partition mechanism, we used different *E*_part_ values from 0.0005 to 0.5 for different bottleneck regimes fitting the expected heteroplasmic sites estimates to the observed number of heteroplasmic sites. The *stratification scheme* parameter defines a set of probabilities of a particular cell to replace any other stem cell when dividing symmetrically. The scheme was designed in a manner that the cells could substitute only the neighboring cells with cells at the end of the rows considered as neighbors (periodic boundaries). Thus, within a specific meristematic layer, all cells could contribute equally to the propagation of mutations.

## Supplementary Material

msae135_Supplementary_Data
