# Supplementary material for: Heteroplasmy Is Rare in Plant Mitochondria Compared with Plastids despite Similar Mutation Rates
Source: Mol Biol Evol. 2024 Jun 27;41(7):msae135. doi: 10.1093/molbev/msae135 (PMC11245704; doi:10.1093/molbev/msae135)
Supplement: msae135_Supplementary_Data [file msae135_supplementary_data.pdf]

Heteroplasmy is rare in plant mitochondria compared to plastids despite similar mutation rates

Supplementary information

Table of Contents

Supplementary information *1*

Supplementary Tables.. *2*

Supplementary Table S1: Replicon copy number in *Z. marina* Finnish clone samples..................2

Supplementary Figures *3*

Supplementary Fig. S1: Haplotype network of *Z. marina* mitochondrial genomes. 3

Supplementary Fig. S2: Haplotype network of *Z. marina* plastid genomes.................................4

Supplementary Fig. S3: Effect of the proportion of symmetric cell divisions (P_{sym}) on the mutation rate (..5

Supplementary Fig. S4: Effects of individual model parameters...6

Supplementary Fig. S5: Effect of the number of stem cells (*N*) on the expected number of heteroplasmic sites. 7

References *8*

Supplementary Tables

Supplementary Table S1: Replicon copy number in *Z. marina* Finnish clone samples. The sampled clone modules are named in accordance with Yu et al. (2020). The replicon copy numbers are estimated for the nucleus (ncDNA/cell), plastids (ptDNA/cell), and mitochondria (mtDNA/cell).

Module	ncDNA/cell	ptDNA/cell	mtDNA/cell
M1	2	239	54
M2	2	153	11
M3	2	198	18
M4	2	285	47
M5	2	153	37
M6	2	182	20
M7	2	227	39
M8	2	218	40
M9	2	301	63
M10	2	183	33
M11	2	215	21
M12	2	180	20
M13	2	224	46
M14	2	202	26
M15	2	194	36
M16	2	316	60
M17	2	240	35
M18	2	202	33
M19	2	229	52
M20	2	215	60
M21	2	241	55
M22	2	181	56
M23	2	202	73
M24	2	207	31

Supplementary Figures

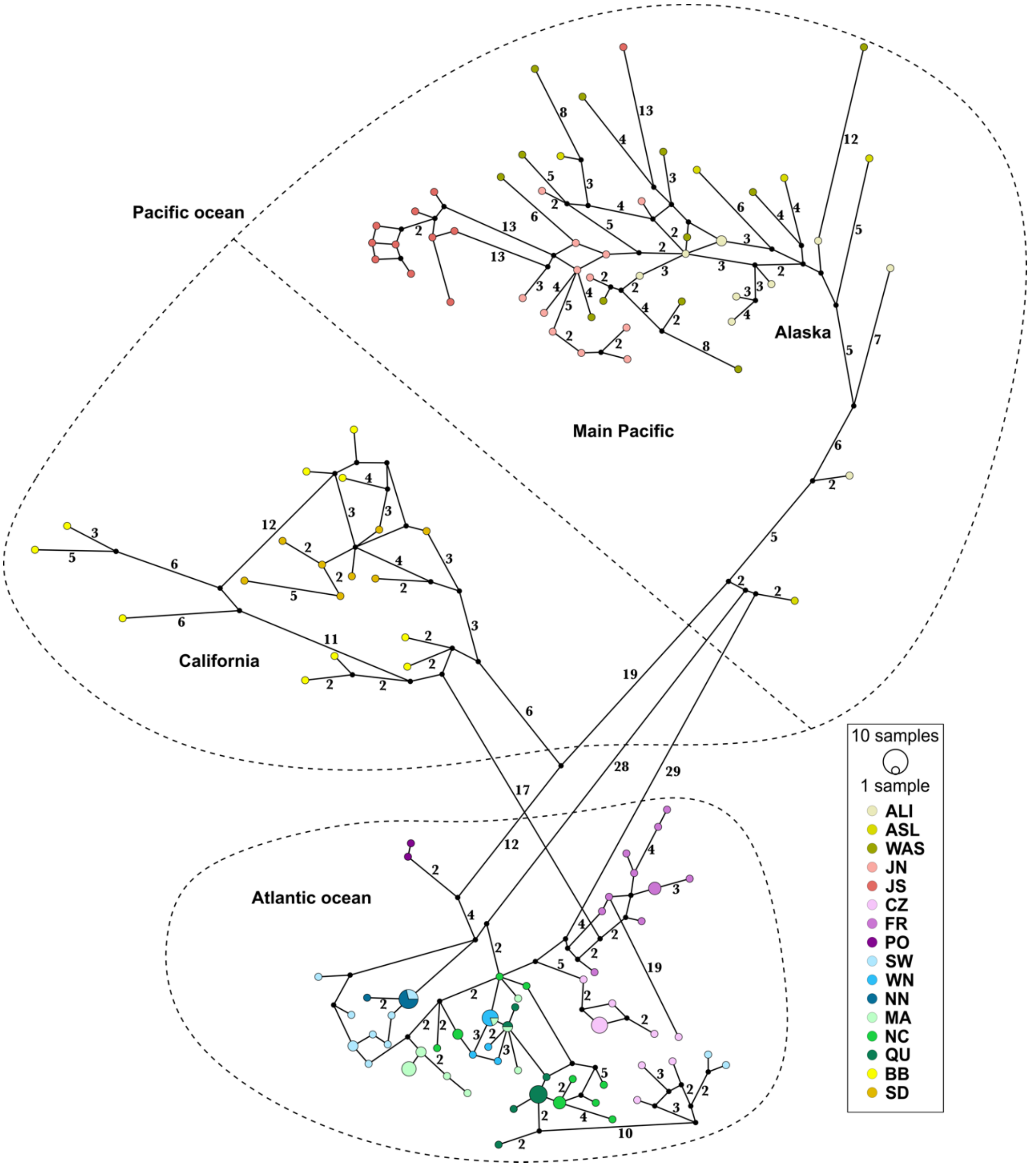

Supplementary Fig. S1: Haplotype network of *Z. marina* mitochondrial genomes. The haplotype network is reconstructed based on fixed SNPs at neutral positions via the TCS Network method implemented in POPART v1.7 with default parameters (Clement et al. 2002; Leigh and Bryant 2015). The haplotypes are colored by the eelgrass population, split-colored circles indicate that a particular haplotype is shared between eelgrass populations, the size of the circle reflects the number of samples. Numbers on the edges show the number of mutation steps if more than one. Main groups of eelgrass populations are marked in accordance with the *Z. marina* phylogeny (Yu et al. 2023). For the population geographic locations see Figure 1. Population abbreviations: California: San Diego, California (SD), Bodega Bay, California (BB); Main Pacific: Washington state (WAS), Japan-North (JN), Japan-South (JS); Main Pacific (Alaska): Alaska-Izembek (ALI), Alaska-Safety Lagoon (ASL); Atlantic ocean: North Carolina (NC), Massachusetts (MA), Quebec (QU), Northern Norway (NN), Sweden (SW), Wales North (WN), Portugal (PO), Mediterranean France (FR), Croatia (CZ).

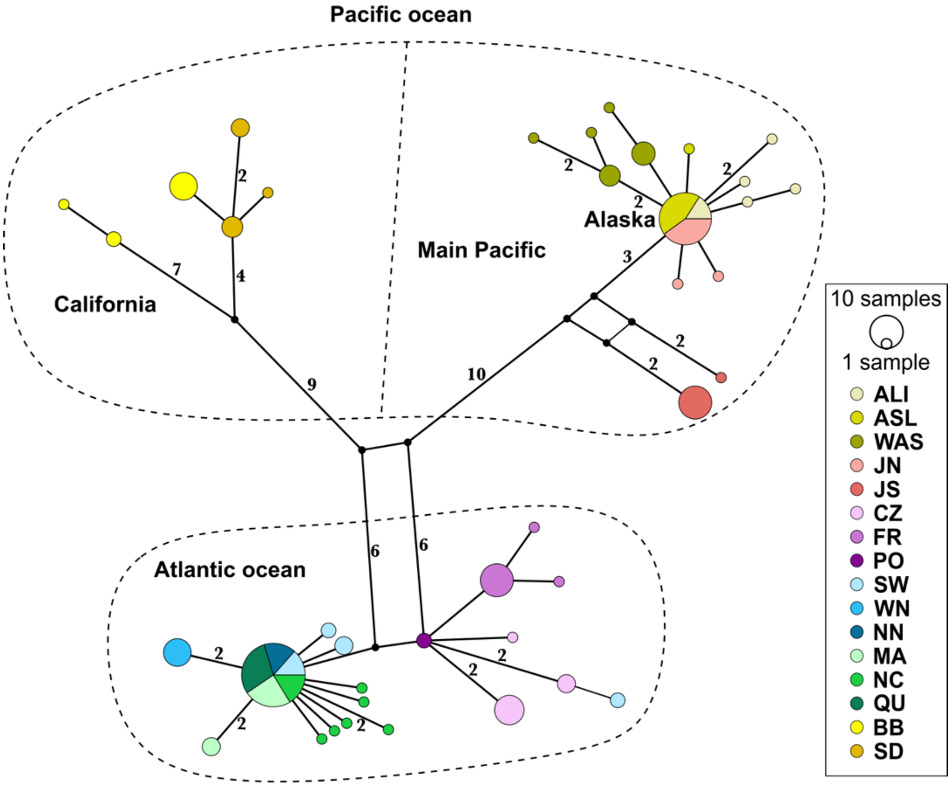

Supplementary Fig. S2: Haplotype network of *Z. marina* plastid genomes. The haplotype network is reconstructed based on fixed SNPs at neutral positions via the Integer Neighbour-Joining method implemented in POPART v1.7 with default parameters (Leigh and Bryant 2015). The haplotypes are colored by the eelgrass population, split-colored circles indicate that a particular haplotype is shared between eelgrass populations, the size of the circle reflects the number of samples. Numbers on the edges show the number of mutation steps if more than one. Main groups of eelgrass populations are marked in accordance with the *Z. marina* phylogeny (Yu et al. 2023). The population abbreviations are the same as on the Supplementary Figure S1.

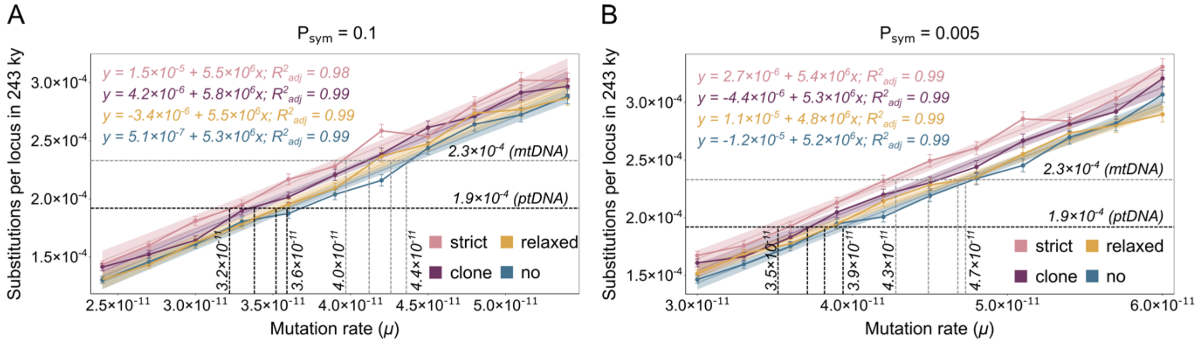

Supplementary Fig. S3: Effect of the proportion of symmetric cell divisions (P_{sym}) on the mutation rate (μ). The number of mitochondrial substitutions per locus in 243,300 years in the *simulation* experiments with $P_{sym} = 0.1$ (A) and $P_{sym} = 0.005$ (B) for different mutation rates (μ). The vertical dashed grey (mitochondrial genome) and black (plastid genome) lines indicate the μ values matching the observed number of accumulated fixed mutations (horizontal dashed lines). The color corresponds to the bottleneck regime, as in Figure 3A legend.

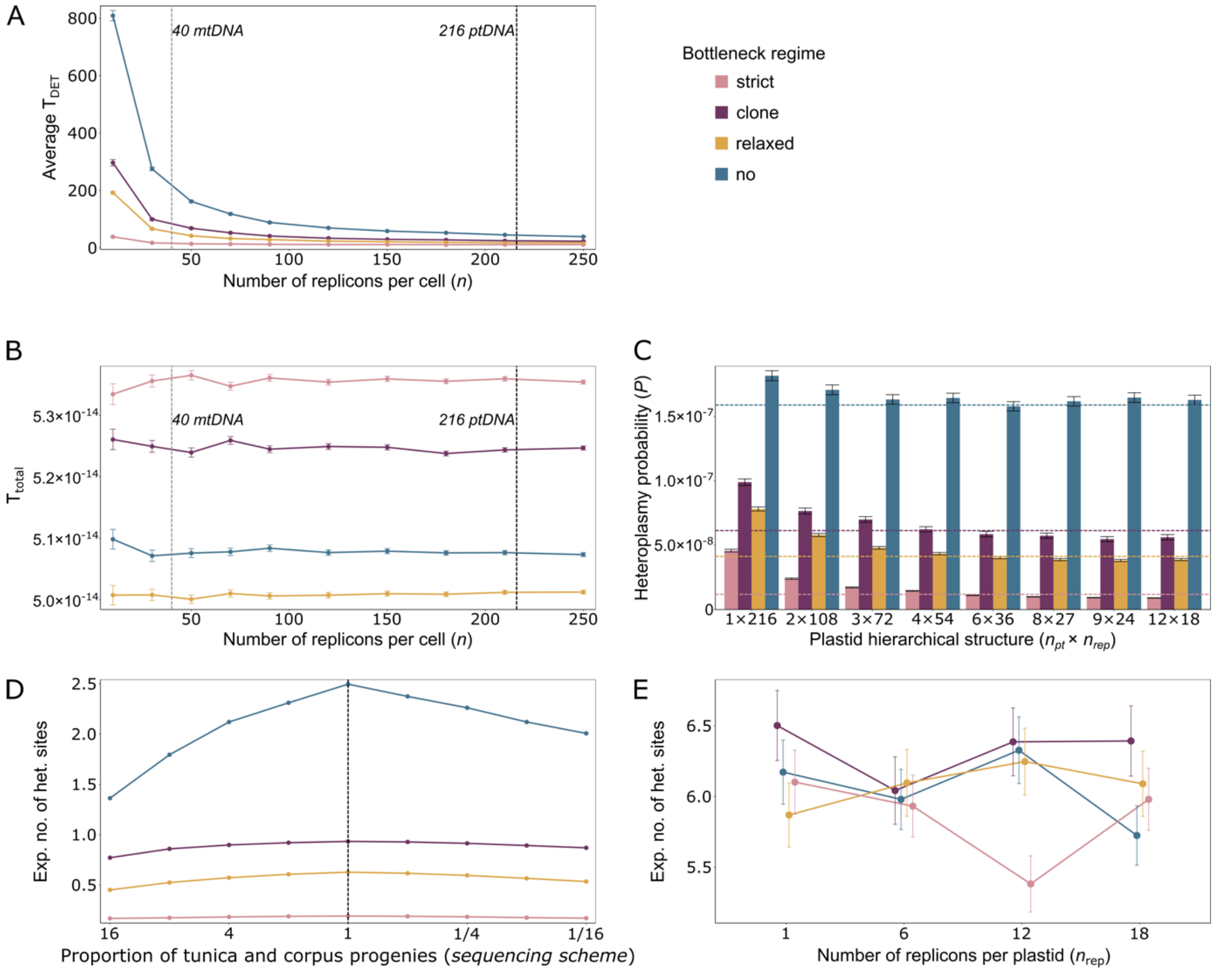

Supplementary Fig. S4: Effects of individual model parameters. a, The average detectable heteroplasmy time in cell divisions (T_{DET}) for different mitochondria-like replicon copy numbers per cell, i.e., with no additional population structure and random segregation. b, The total time in cell divisions (T_{Total}) in the same simulations as in (A) with the constant expected number of fixed mutations $M_{fix} = 500$. The vertical dashed lines correspond to the copy numbers of mtDNA (grey) and ptDNA (black) per cell. c, Effect of the plastid hierarchical structure. The plot depicts the heteroplasmy probability estimated for different combination or ptDNA per plastid (n_{rep}) and plastids per cell (n_{pt}) numbers given a constant number of ptDNA copies per cell ($n = 216$), the mutation rates (μ) that correspond to the estimates for the plastid genome (Figure 2A). The dashed lines reflect the mitochondrial heteroplasmy probability *P* ($n = 40$, mitochondrial μ) calculated for the same simulation parameter set colored by the bottleneck regime. d, Effect of the *sequencing scheme* – the contribution of different apical initials to the sampled tissue. The simulation experiment is conducted for the mitochondrial parameter set. The proportion of progenies is assumed to be equal for stem cells of the same layer, while the proportion of L1 (tunica) layer progenies to L2 (corpus) progenies changes from 16 to 1/16. The expected number of heteroplasmic sites is estimated for 163 *Z. marina* samples. The dashed line reflects the equal number of progenies for all 20 stem cells that was used in other simulations as a default parameter. e, Effect of the number of plastid genome copies per plastid. The number of plastids per cell is $n_{pt} = 12$ in all experiments. The partitioning error (E_{part}) is set in accordance with the bottleneck regimes. The expected number of heteroplasmic sites is estimated for 163 *Z. marina* samples.

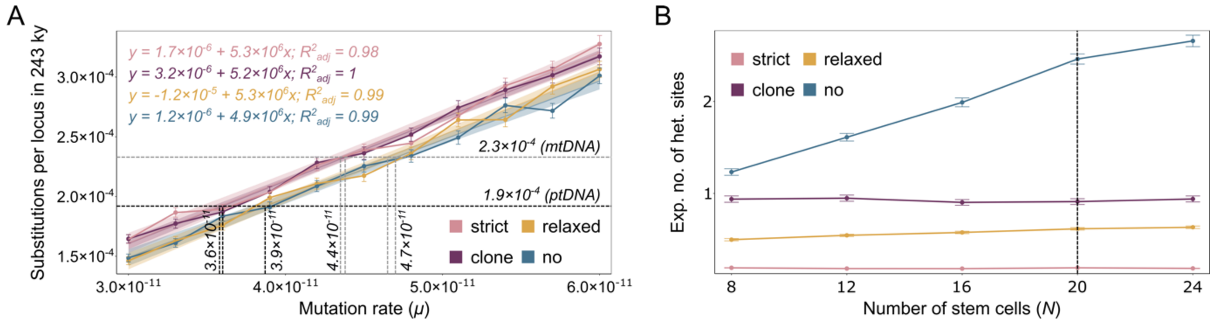

Supplementary Fig. S5: Effect of the number of stem cells (*N*) on the expected number of heteroplasmic sites. a, The number of fixed mitochondrial mutations per base pair in 243,300 years in the *simulation* experiments for different mutation rates (*μ*), proportion of symmetric cell divisions $P_{sym} = 0.01$, $N = 8$ stem cells (four on the tunica layer and four on the corpus layer). The vertical dashed grey (mitochondrial genome) and black (plastid genome) lines indicate the *μ* values matching the observed number of accumulated fixed mutations (horizontal dashed lines). The estimated *μ* values are similar to those estimated for $N = 20$ stem cells (Figure 3A). b, Expected number of heteroplasmic sites in 163 *Z. marina* samples estimated for different number of stem cells (*N*). In each simulation the stem cells were equally distributed between four rows – two in tunica layer and two in corpus layer. The vertical dashed line corresponds to the default number of stem cells in other simulation experiments.

References

Clement M, Snell Q, Walke P, Posada D, Crandall K. 2002. TCS: estimating gene genealogies. In: Proceedings 16th International Parallel and Distributed Processing Symposium. Ft. Lauderdale, FL: IEEE. p. 7 pp. Available from: http://ieeexplore.ieee.org/document/1016585/

Leigh JW, Bryant D. 2015. POPART : full-feature software for haplotype network construction.Nakagawa S, editor. *Methods Ecol Evol* 6:1110–1116.

Yu L, Boström C, Franzenburg S, Bayer T, Dagan T, Reusch TBH. 2020. Somatic genetic drift and multilevel selection in a clonal seagrass. *Nat Ecol Evol* 4:952–962.

Yu L, Khachaturyan M, Matschiner M, Healey A, Bauer D, Cameron B, Cusson M, Emmett Duffy J, Joel Fodrie F, Gill D, et al. 2023. Ocean current patterns drive the worldwide colonization of eelgrass (Zostera marina). *Nat. Plants* 9:1207–1220.
